# Prevalence of readily detected amyloid blood clots in ‘unclotted’ Type 2 Diabetes Mellitus and COVID-19 plasma: a preliminary report

**DOI:** 10.1186/s12933-020-01165-7

**Published:** 2020-11-17

**Authors:** Etheresia Pretorius, Chantelle Venter, Gert Jacobus Laubscher, Petrus Johannes Lourens, Janami Steenkamp, Douglas B. Kell

**Affiliations:** 1 Department of Physiological Sciences, Faculty of Science, Stellenbosch University, Stellenbosch, Private Bag X1, Matieland, 7602 South Africa; 2 Mediclinic Stellenbosch, Suite 104, 1 Elsie du Toit Street, Stellenbosch, 7600 South Africa; 3 PathCare Laboratories, PathCare Business Centre, Neels Bothma Street, N1 City, 7460 South Africa; 4 Department of Biochemistry and Systems Biology, Institute of Systems, Molecular and Integrative Biology, Faculty of Health and Life Sciences, University of Liverpool, Crown St, Liverpool, L69 7ZB UK; 5 The Novo Nordisk Foundation Centre for Biosustainability, Building 220, Kemitorvet, Technical University of Denmark, 2800 Kongens Lyngby, Denmark

**Keywords:** COVID-19, Coagulopathies, Amyloid, Pathologies

## Abstract

**Background:**

Type 2 Diabetes Mellitus (T2DM) is a well-known comorbidity to COVID-19 and coagulopathies are a common accompaniment to both T2DM and COVID-19. In addition, patients with COVID-19 are known to develop micro-clots within the lungs. The rapid detection of COVID-19 uses genotypic testing for the presence of SARS-Cov-2 virus in nasopharyngeal swabs, but it can have a poor sensitivity. A rapid, host-based physiological test that indicated clotting severity and the extent of clotting pathologies in the individual who was infected or not would be highly desirable.

**Methods:**

Platelet poor plasma (PPP) was collected and frozen. On the day of analysis, PPP samples were thawed and analysed. We show here that microclots can be detected in the native plasma of twenty COVID-19, as well as ten T2DM patients, without the addition of any clotting agent, and in particular that such clots are amyloid in nature as judged by a standard fluorogenic stain. Results were compared to ten healthy age-matched individuals.

**Results:**

In COVID-19 plasma these microclots are significantly increased when compared to the levels in T2DM.

**Conclusions:**

This fluorogenic test may provide a rapid and convenient test with 100% sensitivity (P < 0.0001) and is consistent with the recognition that the early detection and prevention of such clotting can have an important role in therapy.

## Background

The standard method for detecting infection with SARS-CoV-2 leading to COVID-19 disease involves a genotypic (PCR) test for the virus on nasopharyngeal swabs, but it is unpleasant, requires specific training, and can have poor sensitivity [1–7]. What would be desirable is a rapid and phenotypic test on the host that indicates the presence, and if possible the severity, of clotting pathologies, which is one of the consequences of infection. Presently, the standard method for this is based on CT chest scans for pneumonia, which have high sensitivity but lower specificity (see [7–10] and below), but this is neither cheap nor universally available.

A poor prognosis for recovery, is linked to various comorbidities, of which Type 2 Diabetes Mellitus (T2DM) is probably the most frequently mentioned comorbidity. It is widely recognised [11–21] that extensive blood clotting has a major role in the pathophysiology of COVID-19 disease severity and progression, yet so can excessive bleeding [22, 23]. The solution to this apparent paradox lies in the recognition [24] that these phases are separated in time: the later bleeding is mediated by the earlier clotting-induced depletion of fibrinogen and of von Willebrand factor (VWF). This first phase of hypercoagulability is accompanied by partial fibrinolysis of the formed clots, and an extent of D-dimer formation that is predictive of clinical outcomes [25]. These features, together with the accompanying decrease in platelets (thrombocytopaenia), leads to the subsequent bleeding. Thus it is suggested that the application of suitably monitored levels of anti-clotting agents in the earlier phase provides for a much better outcome [13, 24]. In addition, dysregulated hemostasis in COVID-19-associated disseminated intravascular coagulation is exacerbated by an inhibition of fibrinolysis, indicating the plasminogen-plasmin-system as a potential target to prevent thromboembolic complications in COVID-19 patients [26]. In addition, patients with COVID-19-associated respiratory failure admitted to the intensive care unit exhibit a hypercoagulable state which is not appreciable on conventional tests of coagulation. Supranormal clot firmness, minimal fibrinolysis, and hyperfibrinogenaemia are key findings [27].

As well as the extent of clotting, including states similar to the life-threatening disseminated intravascular coagulation (DIC) [15], a second issue pertains to its nature. Some years ago, we discovered that in the presence of microbial cell wall components [28, 29], and in a variety of chronic, inflammatory diseases [30–32] (including sepsis [33]), blood fibrinogen can clot into an anomalous, amyloid form [34]. These forms are easily detected by a fluorogenic stain such as thioflavin T, or the so-called Amytracker stains [35]. In all cases, however, these experiments were performed in vitro using relevant plasma, with clotting being induced by the addition of thrombin. In our preliminary experiments this was also the case for plasma from COVID-19 patients, but the signals were so massive that they were essentially off the scale. However, as we report here, the plasma of COVID-19 patients carries a massive load of preformed amyloid clots (with no thrombin being added), and this therefore provides a rapid and convenient test for COVID-19. As the presence of T2DM is a well-known co-morbidity, that significantly decreases survival and a positive outcome for COVID-19 patients, we included such a group in our sample cohort too.

## Methods

### Ethical considerations

Ethical approval for blood collection and analysis of the patients with COVID-19, T2DM and healthy individuals, was given by the Health Research Ethics Committee (HREC) of Stellenbosch University (reference number: 9521). This laboratory study was carried out in strict adherence to the International Declaration of Helsinki, South African Guidelines for Good Clinical Practice and the South African Medical Research Council (SAMRC), Ethical Guidelines for research. Oral consent was obtained from COVID-19 patients to participate in the study. Written consent was obtained from T2DM patients and healthy participants.

### Patient sample

#### Covid-19 patients

20 COVID-19-positive samples (11 males and 9 females) were obtained and blood samples collected before treatment was embarked upon. Blood samples were collected by JS. Platelet poor plasma (PPP) prepared and stored at − 80 °C, until fluorescent microscopy analysis.

#### Type 2 Diabetes Mellitus (T2DM)

Stored Platelet poor plasma samples were randomly selected from our Laboratory’s stored sample repository. 10 age-matched T2DM (6 Males and 4 females), collected in 2018, were used in this analysis.

#### Healthy samples

Our healthy sample was 10 age-matched controls (4 males and 6 females), previously collected and stored in our plasma repository. They were non-smokers, with CRP levels within healthy ranges, and not on any anti-inflammatory medication.

### Lung CT scans

Amongst the COVID-19 patient sample 10 patients were admitted, but stabilized and blood drawn and sent home for observation. Where patients were clinically deemed as moderate or severely ill, CT scans of the patients were performed to determine the severity of the lung pathology. We divided our sample into mild disease (no CT scan) and moderate to severely ill. The CT scan and severity score [36] confirmed moderate to severely ill patients according to the ‘ground glass’ opacities in the lungs.

### Fluorescent Microscopy of patient whole blood and platelet poor plasma (PPP)

A simple fluorescence assay was developed by comparing fluorescent (anomalous) amyloid signals present in PPP from COVID-19 patients, T2DM and those from healthy age-matched individuals, all of whom were studied using PPP that had been stored at −80 °C. On the day of analysis, PPP was thawed and incubated with the dye thioflavin T (ThT; 5 µM final concentration), which detects amyloid-like structures [37]. Following this, the sample was incubated for 30 min (protected from light) at room temperature. PPP smears were then created by transferring a small volume (5 µl) of the stained PPP sample to a microscope slide (similar methods were followed to create a blood smear). A cover slip was placed over the prepared smear and viewed using a Zeiss AxioObserver 7 fluorescent microscope with a Plan-Apochromat 63x/1.4 Oil DIC M27 objective.

For ThT quantification, the excitation was set at 450 to 488 nm and emission at 499 to 529 nm. Unstained samples were also prepared with both healthy and COVID-19 PPP, to assess any autofluorescence. Micrograph analysis was done using ImageJ (version 2.0.0-rc-34/1.5a). The % area of amyloid were calculated using the thresholding method. This method allows a measurement of area of amyloid signal. The RGB images are opened in ImageJ, each image is calibrated by setting the scale (calculated using the image pixel size and the known size of the scale bar). Each image is then converted to black and white (8 bit, this is adjusted under the image type setting). The next step is to threshold the image by adjusting the background intensity to white (255) and then thresholding the now black amyloid signal (in these images between 11 and 15). We used the Huang setting during thresholding. Huang’s method is an optimization method which finds the optimal threshold value by minimizing the measures of fuzziness. The black amyloid area is then analyzed using the analyze particle setting where we use the particle size that is measured from 1 to infinity. The particle size setting allows us to exclude any background signal that might not be true amyloid signal. The area per data per particle size that is generated is then copied into a spreadsheet (see our raw data). Statistical analysis was done using GraphPad Prism 8 (version 8.4.3). Sensitivity and specificity of the data were calculated according to the following calculations:

$$\begin{gathered} {\text{Sensitivity}} = \,{\text{true}}\,{\text{positive}}\,{\text{fraction}}\,{ = }\,\frac{{{\text{true}}\,{\text{positive}} \times 100\% }}{{{\text{true}}\,{\text{positive}}\, + \,{\text{false}}\,{\text{negatives}}}} \hfill \\ {\text{Specificity}} = \,{\text{true}}\,{\text{negative}}\,{\text{fraction}}\,{ = }\,\frac{{{\text{true}}\,{\text{negatives}} \times 100\% }}{{{\text{true}}\,{\text{negatives}}\, + \,{\text{false}}\,{\text{positives}}}} \hfill \\ \end{gathered}$$

## Results

**Fig. 1:**
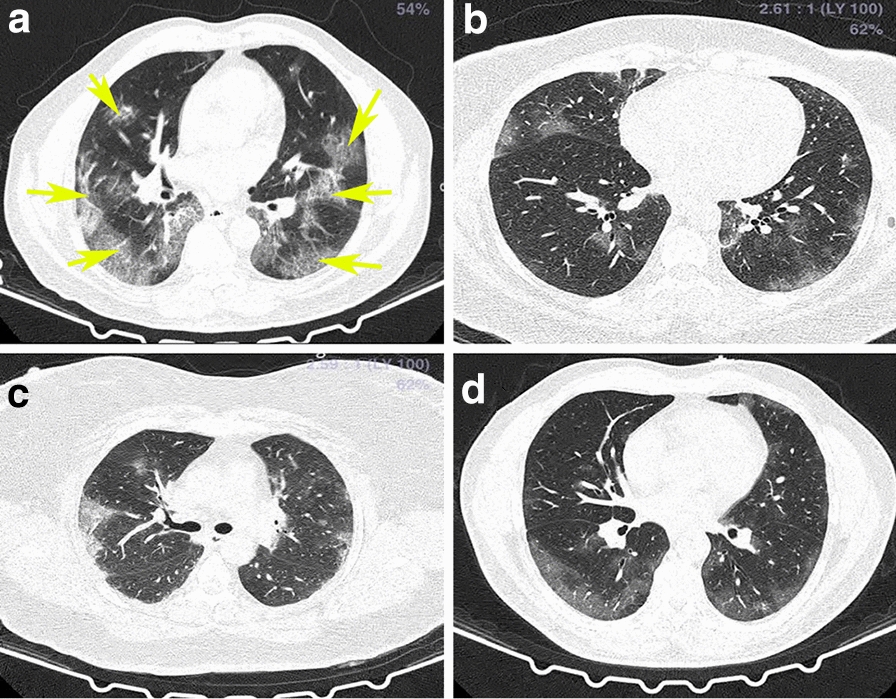
** a**–**d** Representative CT scans of a COVID-19 patient. Yellow arrows show ground glass opacities

Age-matched COVID-19 (average age 49.9y) and healthy individuals (58.8y), and T2DM (62.1y) were used in this analysis (p = 0.06). Platelet poor plasma (PPP) was collected and frozen. On the day of analysis, all PPP samples were thawed and analysed. We also confirmed that the same results are visible in freshly prepared PPP samples. Figure 1 shows representative CT scans of four of the COVID-19 patients. Raw data are shared in https://1drv.ms/u/s!AgoCOmY3bkKHirZOu5YKPlq1x5f1AQ?e=xmWGKm.

**Fig. 2:**
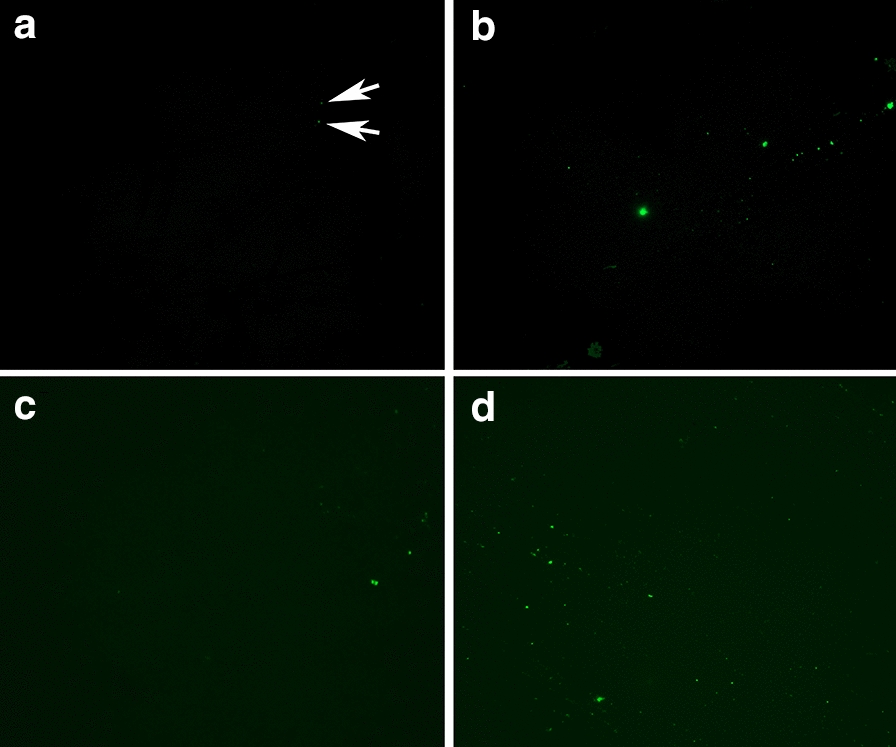
** a**–**d** Representative fluorescence micrographs of platelet poor plasma from healthy individuals. Most signals are very weak, as shown by the arrows in **a**

**Fig. 3:**
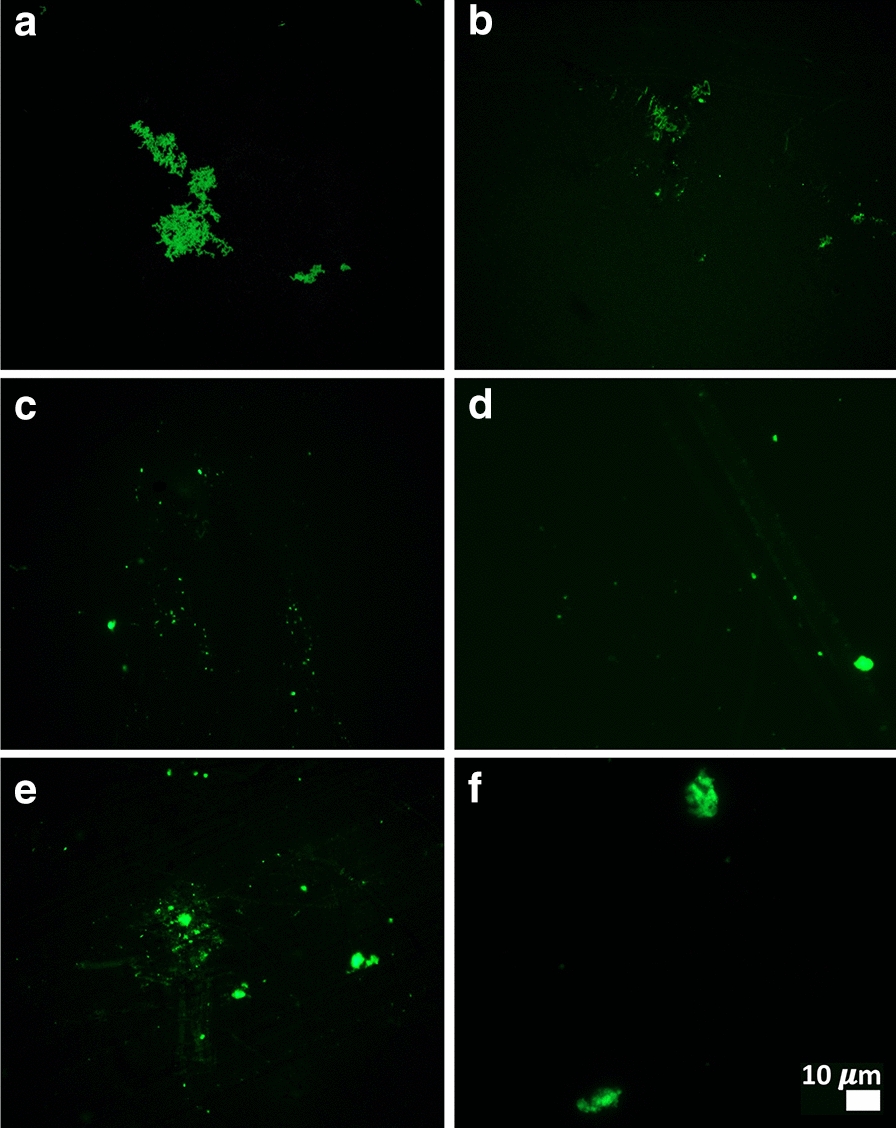
** a**–**f** Representative fluorescence micrographs of platelet poor plasma from Type 2 Diabetes Mellitus (T2DM) patients

**Fig. 4:**
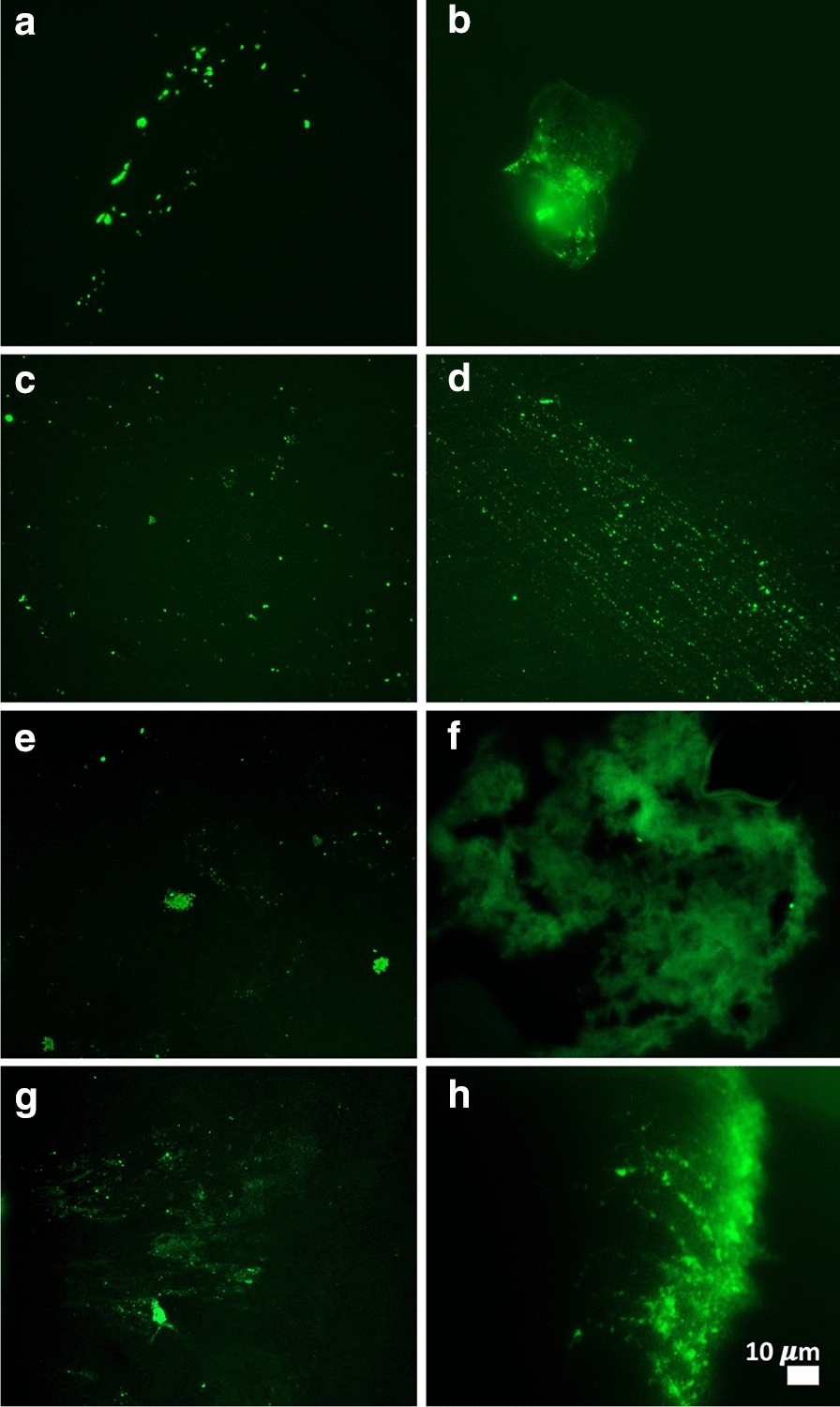
** a**–**h** Representative fluorescence micrographs of platelet poor plasma from COVID-19 patients

**Fig. 5:**
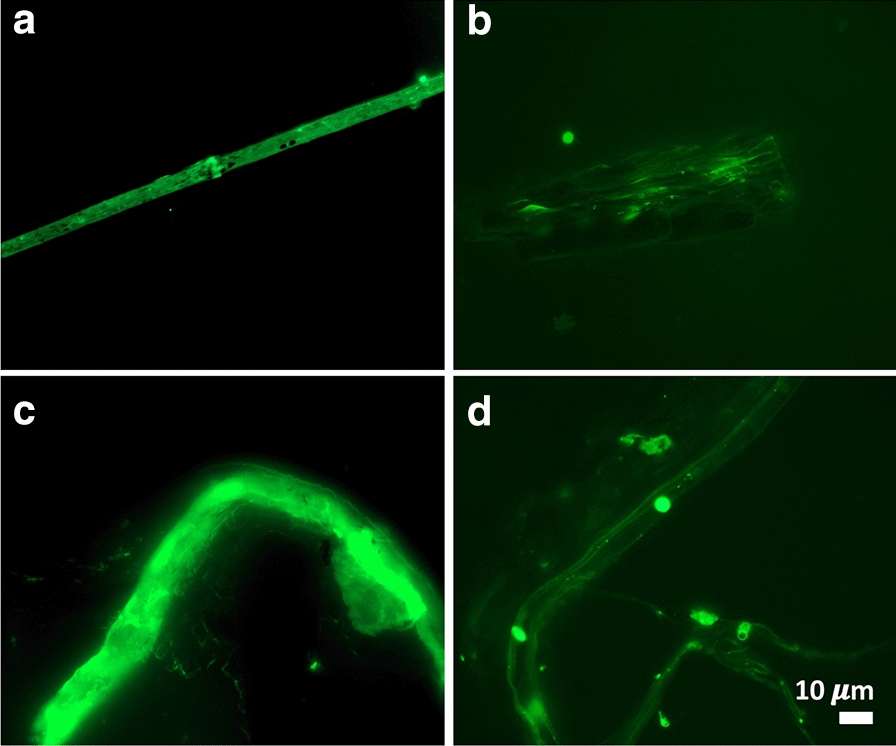
Fibrous or cellular deposits in the plasma smears from COVID-19 patients

**Fig. 6:**
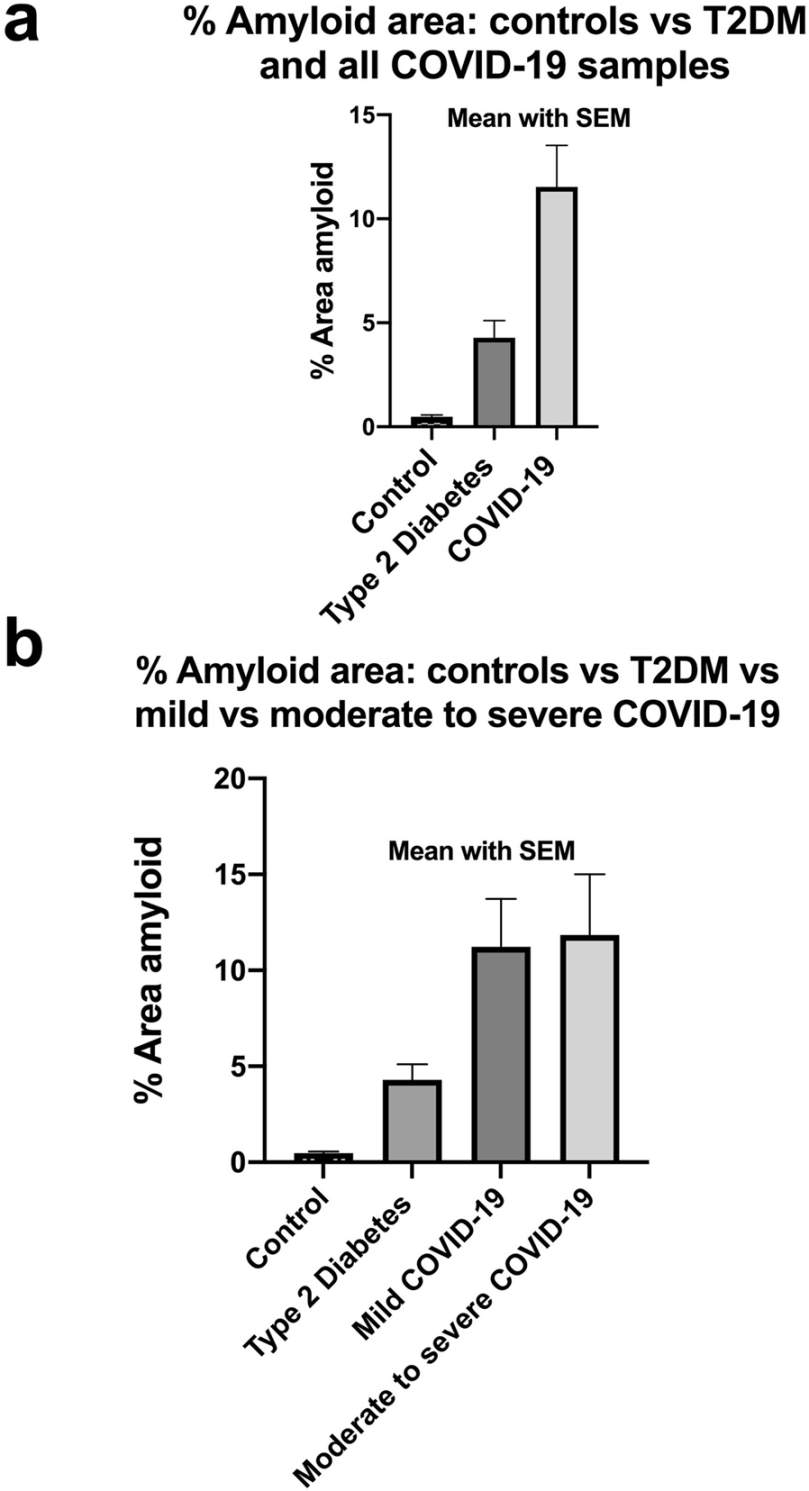
** a**, **b** Amyloid % area in platelet poor plasma smears with mean and SEM (p =  < 0.0001). **a** All controls, Type 2 Diabetes Mellitus (T2DM) and all COVID-19 patients. **b** All controls vs T2DM vs 10 mild and 10 moderate to severely ill COVID-19 patients

Figures 2, 3, 4, 5 show representative fluorescence micrographs of PPP from healthy, T2DM and COVID-19 individuals. In healthy PPP smears (Fig. 2), very little ThT fluorescent signal is visible. In plasma smears from T2DM (Fig. 3), individuals, there were a significant increase in signal, compared to controls, and an even more pronounced increase in signal in COVID-19 individuals (Fig. 4), where abundant amyloid signal is noted. Note that these signals were as received; no thrombin was added to induce clotting. Figure 5 shows the additional presence of fibrous or cellular deposits in the PPP smears of COVID-19 patients. There have been reports of extensive endotheliopathy in COVID-19 patients [38, 39], and these deposits might contribute to this endotheliopathy. Figure 6a and b show box plots of the % area of amyloid signal calculated from representative micrographs of each individual. A nonparametric one-way ANOVA test (Kruskal–Wallis test) between all groups showed a highly significant difference (p =  < 0.0001). However, a Mann–Whitney analysis between the mild and the moderate to severe COVID-19 individuals showed no significant difference (p = 0.554). Amyloid formation in plasma is therefore present in the early stages of COVID-19, when the patients are sufficiently unwell to visit the hospital and in need of stabilization.

### Sensitivity and specificity

Table 1 shows the average % amyloid area for each sample, ranked from lowest to highest values, as well as the sensitivity and specificity calculations. We set the cut-off % amyloid area as 1.3% for controls, and 3.05% for T2DM (see Table 1 and raw data file in shared data link). Using these calculations, the % amyloid area sensitivity and specificity in control versus COVID-19 samples is 85% and 100% respectively, and % amyloid sensitivity and specificity in T2DM versus COVID-19 is 69% and 67%, respectively. Similarly, the % amyloid area sensitivity and specificity for controls versus T2DM is 100% and 100% respectively, suggesting that T2DM is potentially a big confounder. These results suggest that T2DM disease increase the propensity for an individual to develop COVID-19.

**Table 1:** Sensitivity and specificity of % area amyloid (% amyloid was scaled for each sample type from low to high)

Controls	T2DM	COVID-19	Controls vs COVID-19	
0.09%	T2DM Cut-off % amyloid area set as 3.05%		True positives	17 × COVID-19
0.11%			True negatives	10 × Controls
0.18%			False positives	0
0.24%			False negatives	3 × COVID-19
0.36%			Sensitivity	85%
		0.37%	Specificity	100%
0.43%			T2DM vs COVID-19	
0.48%			True positives	9 × COVID-19
0.80%			True negatives	10 × T2DM
		0.81%	False positives	5 × T2DM
0.96%		1.07%	False negatives	4 × COVID-19
1.23%			Sensitivity	69%
Control cut-off % amyloid area set as 1.3%	1.47%		Specificity	67%
			Controls vs T2DM	
	1.86%		True positives	10 × T2DM
	2.12%		True negatives	10 × Controls
	2.73%		False positives	0
	2.84%		False negatives	0
		3.00%	Sensitivity	100%
	3.35%		Specificity	100%
		3.60%		
		3.60%		
		3.64%		
		3.87%		
		4.03%		
	4.98%			
		5.08%		
	5.28%			
	6.73%			
		9.29%		
	9.87%			
		14.39%		
		16.29%		
		18.45%		
		18.69%		
		18.91%		
		21.34%		
		21.79%		
		26.05%		
		36.39%		

## Discussion

Strongly bound up with the coagulopathies accompanying severe COVID-19 disease is the presence of hyperferritinaemia (in cases such as the present it is a cell damage marker [40]) and a cytokine storm, [41–45] which usually occurs in the later phase of the disease [24]. In addition, there has been reports of pulmonary vascular endothelialitis, thrombosis, and angiogenesis in Covid-19 [39]. In addition, excess iron has long been known to cause blood to clot into an anomalous form [46], later shown to be amyloid in nature [28–34]. Increased serum ferritin levels are also known to be present in T2DM [47–50]. These kinds of phenomena seem to accompany essentially every kind of inflammatory disease (e.g. [51]), but the amyloidogenic coagulopathies are normally assessed following the ex vivo addition of thrombin to samples of plasma.

Many clinical features of COVID-19 are unprecedented, and here we demonstrate yet another: the presence in PPP to which thrombin has not been added of amyloid microclots. These microclots are also an pathological feature of PPP from T2DM patients, however there is a significant increase of the microclots in COVID-19 patients. This kind of phenomenon explains at once the extensive microclotting that is such a feature of COVID-19 [11], and adds strongly to the view that its prevention via anti-clotting agents should lie at the heart of therapy. In addition, individuals with T2DM are more prone to develop microclots, due to an increased presence of circulating inflammatory biomarkers that cause hypercoagulability. T2DM patients are therefore predisposed due to their condition. When these individuals then contract SARS-CoV-2, they are already prone to hypercoagulation. This hyperocuagulable predisposition, explains why individuals with T2DM are more prone to develop severe hypercoagulability when diagnosed with COVID-19. Although fluorescence microscopy is a specialized laboratory technique, TEG^®^ is a well-known point of care technique, which is cheap and reliable. Samples can be collected and PPP can be analysed immediately, or frozen and thawed for later analysis. All told, the relative ease of fluorescence microscopy, speed (40 min including 30 min ThT incubation time) and cheapness of the assay we describe might be of significant utility in differentiating COVID-19 from other inflammatory diseases.

Of course this must also be monitored (e.g. via Thromboelastography [52–55]) lest the disease enters its later phase in which bleeding rather than clotting is the greater danger [24]. Although not shown here, an important consideration is that TEG^®^ can be used to study the clotting parameters of both whole blood and PPP. Whole blood TEG^®^ gives information on the clotting potential affected by the presence of both platelets and fibrinogen, while PPP TEG^®^ only presents evidence of the clotting potential of the plasma proteins [52–55].

Point-of-care devices and diagnostics like TEG^®^ are also particularly useful to assess fibrinolysis. In COVID-19 patients, Wright and co-workers reported fibrinolysis shutdown, confirmed by complete failure of clot lysis at 30 min on the TEG^®^ [56]. Thus TEG^®^ can therefore predict thromboembolic events in patients with COVID-19 [56].

## Conclusion

What we have shown here is that the clotting that is commonly seen in COVID-19 patients is in an amyloid form that forms large deposits that might be able to occlude fine capillaries. In addition, these deposits would interfere with fibrinolysis and cause the decreased ability to pass O_2_ into the blood that is such a feature of the disease. As T2DM is a significant comorbidity to COVID-19, exceptional care must be taken when such patients are diagnosed with COVID-19. Consequently, the prevention of coagulopathies must lie at the heart of successful therapies.

## Data Availability

The datasets generated as well as figure micrographs analyzed during the current study are available: The raw data supporting the conclusions of this article will be made available by the authors, without undue reservation. https://1drv.ms/u/s!AgoCOmY3bkKHirZOu5YKPlq1x5f1AQ?e=xmWGKm.
